# Medical Thoracoscopy Performed Using a Flexible Bronchoscope Inserted through a Chest Tube under Local Anesthesia

**DOI:** 10.1155/2009/394817

**Published:** 2009-06-07

**Authors:** Toshinobu Yokoyama, Reiko Toda, Ryusuke Tomioka, Hisamichi Aizawa

**Affiliations:** Division of Respirology, Neurology and Rheumatology, Department of Internal Medicine, Kurume University, 67 Asahi-machi, Kurume 830-0011, Japan

## Abstract

*Background and Objectives*. Many cases of pleural effusion can remain undiagnosed following thoracentesis. We evaluated our own technique for performing thoracoscopy under local anesthesia using a 32 Fr chest tube and a flexible fiberoptic bronchoscope without a rigid thoracoscope for the diagnosis, inspection, and management of patients with pleurisy. *Methods*. Seven patients with pleural effusion who underwent thoracoscopy under local anesthesia using a 32 Fr chest tube and a flexible fiberoptic bronchoscope were retrospectively studied. *Results*. Thoracoscopy was safely performed in the diagnosis and management of pleural effusion in all cases. The visualization of the pleura, diaphragm, and lung using this instrumentation was excellent in comparison to that normally obtained during surgical thoracoscopy. A forceps biopsy of the pleura or diaphragm could therefore be easily and effectively performed. *Conclusion*. This technique is considered to have clinical utility as a diagnostic tool for pleurisy; furthermore, this method is safe, effective and inexpensive, not only for surgeons but also for physicians.

## 1. Introduction

Pleural effusion may arise from a wide range of diseases. However, the diagnosis of this condition may sometimes be difficult. The diagnosis of exudative pleurisy largely depends upon clinical suspicion and laboratory confirmation, by examining the pleural fluid for cytology and adenosine deaminase (ADA) [1]. The diagnosis of pleural effusion can be difficult and this may lead to a delay in treatment. There are many useful tools available for the diagnosis of pleurisy, including a biopsy via thoracoscopy under either general or local anesthesia [2, 3].

## 2. Patients and Method

**Figure 1:**
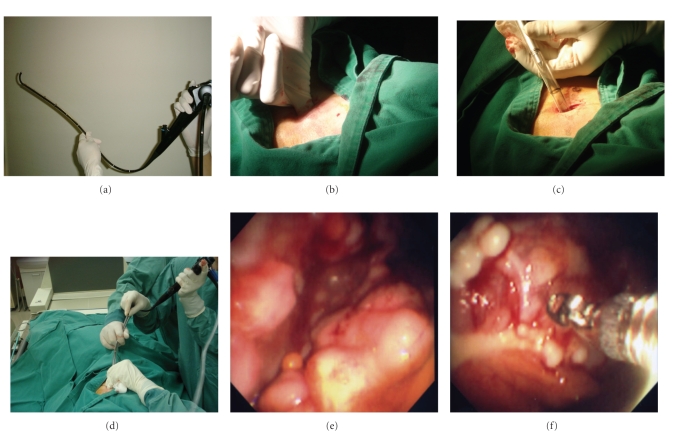
(a) Thoracoscopy using a 32 Fr chest tube and a flexible fiberoptic bronchoscope. The bronchoscope does not bend because of chest tube work as a hard sheath just like having a solid body while it still has a flexible terminal section. (b), (c) chest tube insertion is identical to the insertion of a chest drainage, (d) flexible fiberoptic bronchoscope was inserted into the pleural space thorough the chest tube. (e) visualization of the pleura and diaphragm of a patient with malignant pleural mesothelioma by using this method, (f) a forceps biopsy of the pleura is both effective and easy to perform.

Seven patients (6 males and 1 female, age range from 48 to 88, average 67.1 years old) with pleurisy were examined by thoracoscopy under local anesthesia. These procedures were performed to diagnose the etiology of 3 patients with pleural effusion, and to inspect or manage 4 patients with previously diagnosed pleurisy. An Argyle-type 32 Fr chest tube (trocar catheter, Nippon Sherwood, Tokyo, Japan) and a gas sterilized 5 mm outside diameter flexible fiberoptic bronchoscope, Olympus P240 (Olympus Optical Co. Ltd; Tokyo, Japan) were used. All instruments used in this manipulation were also sterilized with either ethylene oxide gas sterilization (EOG) or another appropriate method. The premedication dose was 15 mg of pentazosine and 0.5 mg of atoropine (atoropin was also administered if necessary). The examination was performed in an endoscopy room. The patient was placed in the lateral decubitus position with the pleural effusion upside. Ultrasonography was performed to determine the entry point. After disinfection with povidone-iodine and local anesthesia with lidocaine, an approximately 1.5 cm chest incision was made. An Argyle-type 32 Fr chest tube was inserted into the pleural space where the chest drainage tube would be inserted (Figures 1(b), 1(c)), and then, a flexible fiberoptic bronchoscope was inserted into the chest tube to approach the pleural space, namely the flexible fiberoptic bronchoscope was inserted into the pleural space through a chest tube (Figure 1(d)). This method requires only one trocar entry point for the fiberscope and for biopsy forceps because the biopsy forceps is used through the channel of the flexible fiberoptic bronchoscope which is done in the same manner as for the manipulation during bronchofiberscopy. After observering the pleura, forceps biopsy specimens of suspicious pleural lesions were taken in the case of undiagnosed pleurisy. During this procedure, the blood pressure, pulse rate, electrocardiogram and continuous oximetry were all monitored. Furthermore, treatment inside the pleural space was performed to destroy any fibrin network formation in the thorax for patients with tuberculosis pleurisy. At the end of the procedure, another Argyle-type chest tube (24 Fr or 22 Fr; Nippon Sherwood, Tokyo, Japan) was inserted instead of a 32 Fr chest tube for few days until pleurodesis was achieved in the case of malignant pleural effusion. The procedures of thoracoscopy under local anesthesia in this study was performed by pulmonologist, not surgeon.

In all cases, no antibiotics were administrated to prevent infection either during or after thoracoscopy because all manipulations were performed aseptically. Informed consent was obtained from all patients.

## 3. Results

All thoracoscopy procedures were performed safely without any accidents or any other serious complications. This procedure was well tolerated and no complications were encountered even with extremely elderly patients. Visualization using this instrumentation was sufficiently accurate to make a diagnosis of pleural lesions. The inspection of the pleura, diaphragm and lung was possible. Regarding the diagnosis of patients with pleural effusion, one patient with malignant mesothelioma (Figures 1(e), 1(f)), one patient with parapneumonic pleural effusion and one patient with myelomatous pleurisy were all successfully diagnosed (myeloma was diagnosed by a biopsy with a Cope needle which was performed simultaneously with thoracoscopy). One patient with adenocarcinoma was confirmed based on endoscopic findings. Three cases of tuberculous pleurisy were visualized. In one of three patients with tuberculous pleurisy after conventional chest tube drainage, thoracoscopy successfully drained the remaining pleural effusion by destroying the fibrinous septal formation in the pleural space. Many reports have so far described that thoracoscopy under local anesthesia can be performed in an endscopy suite, not in an operating room. We also have not experienced and cases demonstrating an infection after this procedure.

## 4. Discussion

Many cases of pleural effusion can remain undiagnosed based on the patient's medical history, and the findings of physical examinations and thoracentesis. The next diagnostic procedure is a percutaneous closed pleural biopsy, using either an Abram biopsy needle or Cope biopsy needle. However, the rate of making a successful diagnosis remains unsatisfactory. After thoracentasis and a closed pleural biopsy, approximately 20% to 30% of patients with pleural effusion remain undiagnosed [4, 5]. Furthermore, sometimes accidents occur during a percutaneous closed pleural biopsy, such as bleeding or a pneumothorax and they can be life-threatening because this examination is performed blind. Recently, thoracoscopy has been performed instead of a percutaneous closed pleural biopsy under general anesthesia using rigid thoracoscopes or under local anesthesia using flexible bronchoscopes [2]. Thoracoscopy can provide a definitive diagnosis or excellent diagnostic yield in comparison with a closed pleural biopsy. Thoracoscopy under local anesthesia using flexible bronchoscopes is a simpler procedure, but they are more difficult to manipulate within the pleural cavity than within the bronchi. Flexible bronchoscopes have several disadvantages in comparison with rigid thoracoscopes, particularly because they do not provide an adequate orientation within the pleural space for smaller biopsies [6, 7]. In addition, it can also provide sufficient materials from forceps biopsy specimens to perform most histological tests including immunohistochemical staining of that for calretinin, WT1, cytokeratin 5/6, and D2-40 in a patient with malignant pleural mesothelioma. Surgical thoracoscopy (video-associated thoracic surgery (VATS)) has several advantages such as the ability to obtain larger biopsy specimens, therapeutic and operative application, and a better control of bleeding in comparison to thoracoscopy under local anesthesia. However, rigid thoracoscopy also has the disadvantages associated with being a more invasive procedure, thus requiring general anesthesia commonly using a double-lumen endotracheal tube or bronchial blocker for selective lung ventilation and therefore it requires the support of an anesthesiologist, a surgical suite, many operative instruments and it must also be performed by surgeons [8]. Local anesthesia thoracoscopy is a less invasive and less expensive, cost benefitical approach to thoracoscopy [9]. The current report demonstrates that local anesthesia thoracoscopy using a 32 Fr chest tube and a flexible fiberoptic bronchoscope is useful for patients with undiagnosed exudative pleural effusions that remained undiagnosed or those requiring the management of pleural diseases. Under local anesthesia, we can talk to the patient during the manipulations by asking such questions as: “Do you feel any dyspnea or pain?” Even when treating elderly patients, we can constantly check to see if there are any problems or complaints. We have thus been able to safely perform this procedure.

Recently, the Olympus LTF-240 semiflexible fiberoptic thoracoscope (Olympus Optical Co Ltd; Tokyo, Japan) has been introduced and evaluated for the diagnosis of pleurisy [10]. This tool combines the features of both instruments, having a solid shaft and a flexible terminal section. In comparison with the LTF-240 and the flexible fiberoptic bronchoscope in local anesthesia thoracoscopy, LTF-240 is better than the flexible fiberoptic bronchoscope for manipulations because the solid body does not bend. When using a 32 Fr chest tube and the flexible fiberoptic bronchoscope does not bend because the chest tube works as a hard sheath and the terminal section is flexible (Figure 1(a)), just like LTF-240. Furthermore, using a 32 Fr chest tube is safe even when either pneumothorax or bleeding occurs. If such complications arise during the procedure for thoracoscopy, then the 32 Fr chest tube can be used as a chest drainage tube directly after removing the flexible fiberoptic bronchoscope. When beginning thoracoscopy, it is easy to drain the pleural effusion with a 32 Fr. chest tube because it is suitable for drainage. In this method of thoracoscopy, the chest tube was not only placed in one direction. By changing either the direction or length of the inserted chest tube, we can, therefore, inspect and perform the biopsy of the pleura from around the apex to diaphragm (Figure 1). In addition, this procedure can also be performed with instruments that are widely available in most clinical situations. Recently, LTF-240 is widely used. From the point of diagnosis and safety of the procedure, the findings of the current report are closely similar to those obtained using LTF, however, number of cases evaluated in this study is small. We therefore consider this new procedure to be safe and effective, and it will continue to become increasingly easier to perform after it has been carried out on a larger number of cases. There are many reports which have so far described thoracoscopy performed by pulmonologists to be a safe and effective modality for the diagnosis of pleural effusion [10, 11]. This method is, therefore, considered to be safe and well tolerated with a minimal degree of discomfort and expense. This method is therefore considered to be useful for surgeons as well as physicians with experience in chest drainage and flexible bronchoscopy.
